# Intervention Effect of Probiotics in Gastric Cancer Patients with Complications of Coronary Heart Disease and Heart Failure

**DOI:** 10.1155/2021/1620891

**Published:** 2021-10-07

**Authors:** Hui Yu, Anqin Dong, Luosha Zhao, Ping Li, Qiujun Zhang, Juying Lu, Ling Li

**Affiliations:** ^1^Department of Cardiology, The First-Affiliated Hospital of Zhengzhou University, Zhengzhou 450052, China; ^2^Department of Rehabilitation Medicine, The Fifth-Affiliated Hospital of Zhengzhou University, Zhengzhou 450052, China; ^3^Department of Medicine, Henan Medical College, Zhengzhou 451191, China; ^4^Department of Nursing, The Fifth-Affiliated Hospital of Zhengzhou, Zhengzhou 450052, China; ^5^Department of Cardiopulmonary Rehabilitation, The Fifth-Affiliated Hospital of Zhengzhou, Zhengzhou 450052, China

## Abstract

**Objective:**

To investigate the characteristics of intestinal flora in patients with gastric cancer complicated by coronary heart disease and heart failure and the guiding value of probiotics intervention for clinical treatment.

**Methods:**

(1) One hundred and sixty-eight gastric cancer patients with complications of coronary heart disease and heart failure from August 2017 to December 2020 were selected as the observation group. A total of 125 patients with coronary heart disease treated at the same time were selected as control group 1, and 89 healthy subjects were selected as control group 2. Fecal samples were retained to extract the total RNA, and high-throughput sequencing was applied to complete the analysis of microbial diversity and structure differences, so as to obtain the biological species information of the specimens. (2) Patients in the observation group were randomly divided into two equal groups of 84 patients, namely, group A and group B. Group A was treated with conventional methods, and group B was combined with probiotics intervention on the basis of group A; then, the differences in the intestinal mucosal barrier between the two groups were compared.

**Results:**

The Chao, ACE, and Simpson index in the observation group were lower than those in control group 1 (*P* < 0.05), and the Shannon index was higher than that in control group 1 (*P* < 0.05). The Chao, ACE, and Shannon index in control group 1 were lower than those in control group 2 (*P* < 0.05), whereas the Simpson index was higher than in control group 2 (*P* < 0.05). The abundance of *Bacteroidetes* in the observation group was lower than that in control group 1 and control group 2 (*P* < 0.05). The abundance of *Firmicutes* was higher than that of control group 1 and control group 2 (*P* < 0.05). Four weeks after treatment, the levels of ET, D-lactic acid, and PCT in the group B were (0.10 ± 0.01), (3.99 ± 0.32), and (0.41 ± 0.10), respectively, which were lower than those in group A (0.19 ± 0.03), (4.51 ± 0.46), and (0.81 0.13).

**Conclusion:**

Gastric cancer patients with complications of coronary heart disease and heart failure are associated with intestinal flora disorder, which may be involved in the occurrence and development of the disease. Probiotics intervention is helpful to repair the intestinal mucosal barrier in patients, which is worthy of popularization and application.

## 1. Introduction

Coronary heart disease is a disease caused by coronary artery stenosis or occlusion of myocardial ischemia, hypoxia, or necrosis. As the course of the disease increases, it can increase the incidence of chronic heart failure [1]. At present, the clinical treatment of coronary heart disease mainly focuses on reducing blood lipids, preventing risk factors, and symptomatic treatment. Although good effects can be obtained, how to choose and take measures to reduce disease morbidity and mortality has become a hot research topic [2]. Gastric cancer is a kind of gastric cancer that originates from gastric mucosal cells, which is mainly clinically characterized by abdominal pain. It usually occurs in people aged 40–70. The incidence rate of men is higher than that of women. Its incidence is mostly related to gastrointestinal dysfunction. Intestinal microbiota refers to the general name of the microorganisms that live in the human gut, which can help the host to complete a variety of physiological and biochemical functions and produce corresponding metabolites [3]. Studies have shown that the host can provide an appropriate environment and necessary nutrition for the intestinal flora, and the intestinal flora can participate in mediating various functions of the human body, provide metabolic nutrients to the host, participate in the promotion of growth and immune regulation, and regulate various functions of the human body [4]. Therefore, strengthening the analysis of the characteristics of the intestinal flora of patients with gastric cancer complicated by coronary heart disease and heart failure can help us understand its role in the pathogenesis of the disease and guide clinical treatment [5, 6]. Probiotics are commonly used as clinical gastrointestinal regulating drugs, regulating intestinal flora, and protecting the intestinal mucosal barrier, but there are few studies on the application of drugs in patients with gastric cancer complicated by coronary heart disease and heart failure [7]. Therefore, this study focused on patients with gastric cancer complicated by coronary heart disease and heart failure to explore the characteristics of the intestinal flora of patients with coronary heart disease combined with heart failure and gastric cancer and the guiding value of probiotic intervention in clinical treatment.

## 2. Materials and Methods

### 2.1. Patients

A total of 168 patients with gastric cancer complicated by coronary heart disease and heart failure from August 2017 to December 2020 at the First-Affiliated Hospital of Zhengzhou University, Zhengzhou, China, were prospectively selected as the observation group. There were 89 males and 79 females, aged (42–84) years, with an average of (61.85 ± 6.61) years; body mass index (BMI) was 18–29 kg/m^2^, average 23.26 ± 4.61 kg/m^2^; coronary heart disease course was 1–9 years, average 4.14 ± 0.61 years; and heart failure course was 1–6 months, with an average of 3.25 ± 0.51 months; patients in the observation group were randomly divided into two groups, A and B, with 84 cases in each group. A total of 125 patients with coronary heart disease who were selected for simultaneous treatment were included in control group 1. There were 67 males and 58 females, aged 43–85 years, with an average of 62.41 ± 6.64 years; BMI was 19–28 kg/m^2^, average 23.31 ± 4.64 kg/m^2^; and duration of disease was 1–10 years, average 4.19 ± 0.67 years; 89 cases of healthy physical examination during the same period were selected as control group 2, with 48 males and 41 females, aged 43–86 years, with average 61.87 ± 6.69 years; BMI was 17–30 kg/m^2^, average 23.86 ± 4.69 kg/m^2^. There was no statistically significant difference in the general information of the patients in each group (*P* > 0.05), and the data were comparable. The study was approved by the ethics committee of the First-Affiliated Hospital of Zhengzhou University, Zhengzhou, China, and the patient's informed consent was obtained.

### 2.2. Inclusion and Exclusion Criteria

#### 2.2.1. Inclusion criteria [8, 9]

(1) Conformed to the diagnostic criteria of coronary heart failure, confirmed by coronary angiography and cardiopulmonary exercise test. (2) Patients in the observation group met the diagnostic criteria for gastric cancer and were diagnosed by pathological examination. (3) All of them could complete the determination of intestinal flora and give probiotics intervention. (4) Complete baseline and follow-up data.

#### 2.2.2. Exclusion criteria

(1) Chronic heart failure caused by valvular disease, hypertension, and cardiomyopathy. (2) Individuals with a mental disorder, abnormal cognitive function, or organic disease. (3) Patients with severely abnormal liver and kidney function accompanied by autoimmune system diseases.

### 2.3. Methods

#### 2.3.1. Analysis of Characteristics of Intestinal Flora

1. Specimen collection and extraction of total DNA of samples
2. After admission, 0.5 g of feces were collected from all three groups and placed in the fecal DNA preservation solution (Shanghai Microcomputer Biotechnology Co., Ltd., Shanghai, China). Total DNA of the samples was extracted according to the instructions (Omega Bio-tek, Norcross, GA, USA), and the concentration and purity of DNA were examined by NanoDrop2000. Subsequently, the DNA was extracted by agarose gel electrophoresis with a concentration of 1% [10].
3. High-throughput sequencing was carried out to complete the analysis of bacterial diversity and species differences, in order to obtain the species information of the specimens. Relevant primers were designed, and the PCR products were recovered by agarose gel with a concentration of 2%. The purified PCR product fragments were amplified by the Illumina MiSeq platform (Illumina, San Diego, USA) to construct a PE 2 300 library. Sequencing was completed with the MiSeq PE300 platform of Illumina (Shanghai Megei Bio-Pharmaceutical Technology Co., Ltd., Shanghai, China), and the obtained data were transferred to the National Biotechnology Information Generality (NCBI) database for processing. TrimMomatic software was applied for quality control during specimen determination [11]. The analysis was performed with Flash software (diversity analysis was carried out from the Chao, ACE, Simpson, Shannon, and the Coverage indexes to detect intestinal flora), and species classification annotation was completed for different sequences. The comparison threshold of the Silva database was set as 70.0% [12].

### 2.4. Intervention in the Observation Group

Both groups were given conventional intervention and conventional symptomatic supportive treatment, such as angiotensin inhibitors Digitalium and diuretics. Group A was utilized conventional methods of treatment. Metoprolol Succinate Sustained-release Tablets (AstraZeneca, Inc., National Drug License: J20150044, specification: 47.5 mg) 95 mg orally every time, once a day, and Trimetazidine Dihydrochloride Tablets (Shanxi C&Y Pharmaceutical Group Co., Ltd., National Drug License: H20123233, specification: 20 mg) 20 mg orally every time, 3 times a day for 4 weeks (1 course of treatment), were given. The treatment of group B is combined with probiotic intervention on the basis of group A. *Bifidobacterium longum*, *Lactobacillus acidophilus*, and *Enterococcus faecalis* Capsules (Shenzhen Xinwanze Pharmaceutical Co., Ltd., National Drug License: S19980004, specification: 0.5 g, containing live bacteria number ≥1.0 × 10^7^ CFU) 2 tablets orally were given in warm water for 30 min after meals, twice a day for 4 weeks (1 course) [13].

### 2.5. Observation Indicators

(1) The Chao, ACE, Simpson, Shannon, and Coverage indexes of the three groups were recorded to analyze intestinal flora diversity. (2) Routine microflora determination was completed in all three groups, and the characteristics of intestinal microflora were analyzed for the level of phylum, class, and genus. (3) 3 mL of peripheral fasting blood was taken from the two groups before treatment and the day after treatment. ELISA assay was performed to determine the patient's endotoxin (ET) and D-lactic acid levels, and the chemiluminescence method was applied to determine procalcitonin (PCT) level, to assess the intestinal mucosal barrier.

### 2.6. Statistical Analysis

SPSS24.0 software was used to process these data. A chi-square test was performed, and *n* (%) was utilized to represent the enumeration data. ET, PCT, and other measurement data were all in line with normal distribution. The *P* < 0.05 was statistically significant.

## 3. Results

### 3.1. Comparison of the Diversity of Intestinal Flora among the Three Groups

The Coverage index of the three groups showed no statistical significance (*P* > 0.05). The Chao, ACE, and Simpson index in the observation group were lower than those in control group 1 (*P* < 0.05), whereas the Shannon index was higher than that in control group 1 (*P* < 0.05). The Chao, ACE, and Shannon index in control group 1 were lower than those in control group 2 (*P* < 0.5), whereas the Simpson index was higher than that in control group 2 (*P* < 0.05), as shown in Table 1.

### 3.2. Analysis of Intestinal Flora Characteristics of the Three Groups

**Figure 1:**
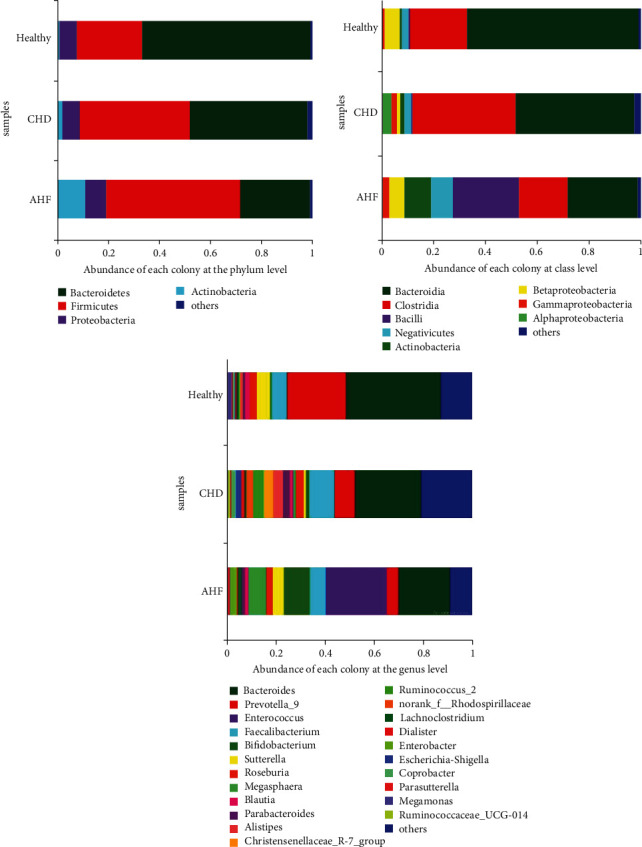
Characteristic analysis of the intestinal flora of the three groups (in phylum, class, and genus level).

The abundance of *Bacteroidetes* and *Firmicutes* in the three groups was statistically significant (*P* < 0.05), respectively. The abundance of *Bacteroidetes* in the observation group was lower than that in control group 1 and control group 2 (*P* < 0.05), respectively. The abundance of *Firmicutes* was higher than that of control group 1 and control group 2 (*P* < 0.05), respectively. The abundance of *Proteobacteria* and *Actinobacteria* in the three groups was not statistically significant (*P* > 0.05), as shown in Figure 1.

### 3.3. Comparison of the Intestinal Mucosal Barrier between the Two Groups

Before treatment, there was no statistical significance between the two groups (*P* > 0.05). Intestinal mucosa was improved significantly in both groups 4 weeks after treatment. The levels of ET, D-lactic acid, and PCT in group B at 4 weeks after treatment were lower than those in group A (*P* < 0.05), as shown in Table 2.

## 4. Discussion

Numerous studies have shown that intestinal flora is involved in the occurrence and development of various diseases, including cardiovascular system disorders [14, 15]. Coronary atherosclerosis is an independent risk factor for coronary heart disease. It is a chronic vascular inflammatory disease and can participate in different stages of plaque, from adhesion molecules to white blood cells to metalloproteinases, causing digestion of fibrous cap and increasing plaque instability [16]. Gastric cancer is a malignant tumor originating from gastric mucosal epithelium, and its incidence has obvious regional differences. In China, it tends to occur on the eastern coast and the northwest. In addition, with the change of dietary structure and the increase of work pressure and other reasons such as *Helicobacter pylori*, gastric cancer tends to be younger. More and more studies have shown that changes in intestinal flora composition and function will cause intestinal flora imbalance, which can accelerate the occurrence of cardiovascular diseases [17]. In this study, the Chao, ACE, and Simpson index of the observation group were lower than those of the control group 1. The Shannon index was higher than that in control group 1. The Chao, ACE, and Shannon index in control group 1 were lower than those in control group. The Simpson index was higher than that in control group 2. The abundance of *Bacteroidetes* in group B was lower than that in group A, while the abundance of *Firmicutes* was higher than that in group A. According to these pieces of evidence, gastric cancer patients with complications of coronary heart disease and heart failure are accompanied by obvious intestinal flora disorder, which can be directly involved in the pathological process of the disease. It may be due to the intestinal flora disorder of patients, which can cause the increase of the level of inflammatory factors in the body through its metabolites, thus accelerating the course of atherosclerosis and heart failure and affecting cardiovascular diseases. From the analysis of intestinal flora characteristics of gastric cancer patients with complications of coronary heart disease and heart failure, the intestinal flora of patients contains a higher *Firmicutes* and lower *Bacteroidetes*, mainly because *Bacteroidetes* can participate in the metabolism of a variety of substances, promote the formation of intestinal mucosal blood vessels, and maintain the balanced metabolism of normal intestinal flora [18]. However, in gastric cancer patients with complications of coronary heart disease and heart failure, there are fewer beneficial bacteria that can maintain the intestinal metabolism, which can aggravate the onset in patients [19].

These shreds of evidence have revealed that intestinal flora disturbance can directly participate in the occurrence of coronary heart disease combined with heart failure and gastric cancer patients, being harmful to patients. Probiotics are a commonly used microecological preparation. After oral supplementation of intestinal beneficial bacteria, the beneficial bacteria attach to the intestinal mucosa, then grow, and proliferate in large numbers, which can competitively antagonize the colonization and growth of pathogenic bacteria in the intestinal tract, thus promoting the recovery of normal intestinal flora, helping patients to rebuild and maintain the balance of intestinal flora, and helping patients to construct the intestinal barrier [20]. Previous studies have shown that probiotics can stimulate the local lymphoid tissues of intestinal mucosa, activate the local defense function, and enhance the anti-infection ability of the gastrointestinal tract [21]. In this study, the levels of ET, D-lactic acid, and PCT in group B were lower than those in group A at 4 weeks after treatment, suggesting probiotics for gastric cancer patients with complications of coronary heart disease and heart failure can help to build the intestinal barrier, correct the intestinal flora disorder patients, and reduce the aggravation of the disease caused by intestinal flora. Therefore, the determination of intestinal flora in gastric cancer patients with complications of coronary heart disease and heart failure should be strengthened in clinical practice, and timely probiotics intervention should be given to abnormal patients to promote their recovery.

In conclusion, gastric cancer patients with complications of coronary heart disease and heart failure are often accompanied by intestinal flora disorder, which may be involved in the occurrence and development of various disorders. Probiotics intervention is helpful to repair the intestinal mucosal barrier in patients, which is worthy of popularization and application.

## Figures and Tables

**Table 1:** Comparison of intestinal flora diversity among the three groups ($\bar{x}\pm s$).

Group	Cases	Chao	ACE	Simpson	Shannon	Coverage

Observation group	168	100.61 ± 10.83^#*∗*^	101.21 ± 14.32^#*∗*^	0.10 ± 0.04^#*∗*^	2.59 ± 0.56^#*∗*^	1.04 ± 0.21
Control group 1	125	120.52 ± 18.53^#^	112.59 ± 17.53^#^	0.28 ± 0.09^#^	2.15 ± 0.41^#^	1.06 ± 0.24
Control group 2	89	170.59 ± 24.62	159.74 ± 21.41	0.14 ± 0.06	3.20 ± 0.63	1.03 ± 0.20

Vs. control group 2, ^#^*P* < 0.05; vs. control group 1, ^∗^*P* < 0.05.

**Table 2:** Comparison of the gastrointestinal mucosal barrier between the two groups ($\bar{x}\pm s$).

Group		ET (EU/mL)	D-lactate (*μ*g/mL)	PCT (*μ*g/L)

Group B(*n* = 84)	Before treatment	0.25 ± 0.04	6.42 ± 0.61	0.96 ± 0.13
	4 weeks after treatment	0.10 ± 0.01^#*∗*^	3.99 ± 0.32^#*∗*^	0.41 ± 0.10^#*∗*^

Group A(*n* = 84)	Before treatment	0.26 ± 0.05	6.43 ± 0.62	0.98 ± 0.15
	4 weeks after treatment	0.19 ± 0.03^*∗*^	4.51 ± 0.46^*∗*^	0.81 ± 0.13^*∗*^

Vs. group A, ^#^*P* < 0.05; vs. before treatment, ^∗^*P* < 0.05.

## Data Availability

The data used to support the findings of this study are available from the corresponding author upon request.
